# Lesion of the rostromedial tegmental nucleus increases voluntary ethanol consumption and accelerates extinction of ethanol-induced conditioned taste aversion

**DOI:** 10.1007/s00213-016-4406-7

**Published:** 2016-08-23

**Authors:** Chandni Sheth, Teri M. Furlong, Kristen A. Keefe, Sharif A. Taha

**Affiliations:** Department of Pharmacology and Toxicology, University of Utah, 30 South 2000 East, Salt Lake City, UT 84112-5820 USA

**Keywords:** Lateral habenula (LHb), Rostromedial tegmental nucleus (RMTg), Ethanol, Aversion, Reward

## Abstract

**Rationale:**

Ethanol has rewarding and aversive properties, and the balance of these properties influences voluntary ethanol consumption. Preclinical and clinical evidence show that the aversive properties of ethanol limit intake. The neural circuits underlying ethanol-induced aversion learning are not fully understood. We have previously shown that the lateral habenula (LHb), a region critical for aversive conditioning, plays an important role in ethanol-directed behaviors. However, the neurocircuitry through which LHb exerts its actions is unknown.

**Objective:**

In the present study, we investigate a role for the rostromedial tegmental nucleus (RMTg), a major LHb projection target, in regulating ethanol-directed behaviors.

**Methods:**

Rats received either sham or RMTg lesions and were studied during voluntary ethanol consumption; operant ethanol self-administration, extinction, and yohimbine-induced reinstatement of ethanol-seeking; and ethanol-induced conditioned taste aversion (CTA).

**Results:**

RMTg lesions increased voluntary ethanol consumption and accelerated extinction of ethanol-induced CTA.

**Conclusions:**

The RMTg plays an important role in regulating voluntary ethanol consumption, possibly by mediating ethanol-induced aversive conditioning.

## Introduction

Drugs of abuse, including ethanol, have both rewarding and aversive properties (Verendeev and Riley 2013), and the relative balance of these properties importantly influences levels of voluntary drug intake (Riley 2011). The aversive effects of ethanol include nausea, motor impairment, sedation, and hangover-like effects (Schulteis and Liu 2006). In humans, ethanol ingestion results in lower sedative responses in heavy vs. light drinkers (King et al. 2011). Further, for heavy drinkers, decreased levels of aversive sedation are predictive of increased ethanol-binge frequency, as well as increased likelihood of developing an alcohol-use disorder. These results show that in humans, decreased sensitivity to the aversive effects of ethanol is strongly associated with increased ethanol intake (King et al. 2011; King et al. 2014).

Multiple lines of evidence from rodent studies suggest that the aversive effects of ethanol act to limit voluntary intake. Voluntary ethanol consumption in mouse strains is inversely correlated with ethanol-induced conditioned taste aversion (CTA) (Belknap et al. 1993; Phillips et al. 2005; Green and Grahame 2008; Cunningham et al. 2009). Similarly, sex-dependent differences in adolescent rats’ voluntary consumption of ethanol are inversely related to ethanol-induced CTA (though this relationship was not observed in adult rats; Vetter-O’Hagen et al. 2009). Further, alcohol-preferring (P) rats do not decrease preference for ethanol after involuntary ethanol pre-exposure, in contrast to wild-type Sprague-Dawley rats (Rezvani et al. 2010). P rats also show attenuated ethanol-induced CTA relative to alcohol-non-preferring (NP) rats (Froehlich et al. 1988). These data suggest that decreased sensitivity to aversive effects of ethanol may contribute to high ethanol intake in P rats. Notably, the measures of aversion included above are heterogeneous (and likely have distinct underlying mechanisms), suggesting that ethanol-induced aversion may be broadly implicated in regulating voluntary intake. Together, these preclinical and clinical studies provide evidence suggesting that attenuated aversion to ethanol may contribute to high levels of voluntary ethanol consumption.

Characterizing the neural circuits underlying ethanol’s aversive effects is therefore relevant for understanding mechanisms underlying ethanol intake and vulnerability to addiction. The lateral habenula (LHb) is an epithalamic brain region implicated in regulating aversive conditioning (Matsumoto and Hikosaka 2007, 2009). The role of the LHb in aversion learning is importantly mediated through inhibition of midbrain dopamine (DA) neurons (Christoph et al. 1986; Ji and Shepard 2007). This inhibition is mediated through a disynaptic relay, in which excitatory LHb projections target GABAergic neurons in the midbrain rostromedial tegmental nucleus (RMTg, also called the tail of the ventral tegmental area [tVTA]) which in turn target and inhibit ventral tegmental area (VTA) DA neurons (Jhou et al. 2009b; Kaufling et al. 2009, 2010; Hong et al. 2011). Stimulation of either the LHb or the RMTg inhibits firing in midbrain DA neurons (Christoph et al. 1986; Lecca et al. 2012), suggesting that activity in the LHb-RMTg pathway acts as a “brake pedal” on DA neuron firing (Barrot et al. 2012). Consistent with this idea, activation of the LHb-RMTg circuit drives aversion learning (Stamatakis and Stuber 2012), and lesion of this circuit abolishes drug-induced learned avoidance (Jhou et al. 2013).

Our lab has previously shown that lesions of the LHb increase voluntary ethanol consumption in rats trained in an intermittent ethanol access (IEA) paradigm (Carnicella et al. 2014; Haack et al. 2014). Specifically, LHb-lesioned rats escalated intake more rapidly than sham-lesioned controls over a period of weeks and voluntarily consumed roughly 50 % more ethanol in 24-h sessions after 8 weeks of IEA. Operant self-administration of ethanol was also significantly increased in LHb-lesioned animals. Further, LHb lesions blocked yohimbine-induced reinstatement of ethanol-seeking. Interestingly, the changes in voluntary ethanol consumption in LHb-lesioned animals were accompanied by an attenuation of ethanol-induced CTA. Consistent with these findings, a more recent report has shown that pharmacological inactivation of the LHb eliminates ethanol-induced place aversion (Zuo et al. 2015). While these results are correlative, they raise the interesting possibility that increased voluntary ethanol intake in LHb-lesioned rats results from loss of ethanol-induced aversion learning mechanisms.

The neural circuits through which the LHb modulates voluntary ethanol intake and ethanol-induced CTA are unknown. Given the robust efferent projection from the LHb to the RMTg and role of the RMTg in aversion-driven behaviors, we hypothesized that the RMTg also plays an important role in regulating ethanol-directed behaviors. In the current set of experiments, we studied voluntary ethanol consumption, operant responding, yohimbine-induced reinstatement, and ethanol-induced CTA in RMTg- and sham-lesioned rats. Our results provide strong evidence of a role for the RMTg in regulation of voluntary ethanol intake and extinction of ethanol-induced CTA. These results, combined with our previous findings (Haack et al. 2014), suggest that the LHb and the RMTg play tightly coupled roles in regulating voluntary ethanol intake, possibly by mediating ethanol-induced aversive conditioning.

## Materials and methods

### Subjects

Fifty-seven male Long-Evans rats (300–350 g on receipt; Charles-River, Wilmington, MA) were used in the present study. Rats were single-housed in Plexiglas tub cages and maintained on a 12-h (h) light/dark cycle. Ad libitum access to food and water was available at all times except during conditioned taste aversion experiments (see below for details). All procedures occurred in the light cycle (12:12 h), with lights on at 6 AM unless otherwise stated. All procedures used were approved by the University of Utah Animal Care and Use Committee and carried out in accordance with the *Guide for the Care and Use of Laboratory Animals* (8th edition). Table 1 shows timeline of behavioral training and testing and final number of rats used in each experiment.

**Table 1:** Summary of experimental groups, timeline of procedures, and final number of rats used in each experiment

Rat group	Number of sham rats	Number of RMTg-lesioned rats	Experiments
1	10	9	(1) Intermittent ethanol access (2) Operant self-administration, extinction, and reinstatement (3) Ethanol-induced conditioned taste aversion (CTA)
2	6	6	(1) Intermittent ethanol access (2) Blood ethanol concentration (3) Quinine preference (4) Saccharin preference
3	8	7	(1) Intermittent ethanol access (2) Blood ethanol concentration (3) Quinine preference (4) Saccharin preference
4	5^a^ 4	2^b^ 3	(1) Intermittent ethanol access (2) Ethanol-induced conditioned taste aversion (CTA)

Numbered experiments indicate the order in which experiments within each group were carried out. In group 4, superscript letter a denotes one sham-lesioned rat died after IEA yielding *n* = 4 for CTA and superscript letter b denotes one RMTg-lesioned rat was removed from IEA analysis [*n* = 2] because it had negligible ethanol intake but retained in the CTA analysis [*n* = 3])

### Drugs

Ethanol solutions were prepared in filtered tap water to a concentration of 20 % (*v*/v) for use in the intermittent ethanol access (IEA) paradigm. All other drugs were purchased from Sigma-Aldrich (St. Louis, MO). Saccharin and quinine solutions were prepared in distilled water. Yohimbine was prepared at a concentration of 4 mg/ml in distilled water.

### Excitotoxic lesions of the RMTg

Excitotoxic lesion techniques were used to ablate the RMTg to minimize damage to fibers of passage. Surgical procedures were conducted under isoflurane anesthesia (5 % induction, 2 % maintenance). Neo-Predef (a topical anesthetic), buprenorphine (0.06 mg/kg, intraperitoneal [IP]), and penicillin (3 × 108 units/kg, intramuscular [i.m.]) were administered for analgesia and to prevent infection. Rats were placed in a flat-skull position in a stereotaxic apparatus, the skull exposed, and burr holes drilled above the target region. Bilateral excitotoxic lesions were produced using quinolinic acid (0.4 μl of 0.12 M solution in each hemisphere). Infusions were made via a 31 gauge needle connected to polyethylene tubing (PE50) attached to a 1-μl glass Hamilton (Reno, NV) syringe on a Harvard 2000 micro-infusion pump (Harvard Apparatus, Holliston, MA). For each infusion, a volume of 0.4 μl was injected at a rate of 0.2 μl/min, with the needle left in place for an additional 2 min to allow for diffusion. RMTg coordinates were AP:−7.1 mm, ML: ±1.2 mm, and DV: −8.0 mm, relative to bregma (Paxinos and Watson 2007). For sham lesions, the needle was lowered 1 mm dorsal to the RMTg, but no infusion was made.

#### Intermittent ethanol access

Voluntary ethanol consumption was measured for 8 weeks in a two-bottle choice IEA paradigm in 57 rats (29 sham- and 28 RMTg-lesioned). Data from four RMTg-lesioned rats were discarded due to misplaced lesions (2 rats), negligible ethanol intake (1 rat), and mortality (1 rat). Data from 29 sham- and 24 RMTg-lesioned rats were thus included in our analyses.

In the IEA paradigm, rats were given 24-h access to two bottles in their home-cages on alternate weekdays. One bottle contained 20 % ethanol (*v*/*v*) in tap water and the other contained tap water. On Monday, Wednesday, and Friday of each week, the bottles were weighed and placed in the home cages at 9 AM, and then removed and weighed at 9 AM the following day so that total consumption was recorded for each 24-h session. The position of each bottle was switched on successive sessions to minimize side preferences. Ad lib water was available at all times. Food was available ad lib at all times, and food intake and body weight were measured weekly for all rats. In analyzing ethanol intake, total ethanol intake was normalized to body weight (g/kg/24 h), and ethanol preference was calculated (ethanol intake/total fluid (water + ethanol) intake).

#### Taste preference: two-bottle choice for saccharin and quinine solution

Taste preference and taste aversion were assessed using two-bottle choice paradigms comparing the intake of water to that of saccharin and quinine solutions, respectively, in a subset of rats (14 sham- and 13 RMTg-lesioned). Quinine intake was measured first (6 sessions) followed by saccharin intake (6 sessions). Each session consisted of a 48-h period in which two bottles were provided in the home-cage. One bottle contained distilled water, and the second bottle contained either quinine or a saccharin solution. Tastant concentrations increased across sessions (0.001, 0.003, 0.01, 0.03, 0.1, and 0.3 mM concentrations for successive quinine sessions; 0.01, 0.05, 0.1, 0.5, 1, and 5 mM concentrations for successive saccharin sessions). Consumption was recorded at 24-h intervals, and the side of the bottle was switched at this time to minimize side preferences. Quinine and saccharin preference for each concentration (i.e., each 48-h period) was calculated by averaging the intake for the two 24-h periods and then dividing by the average total fluid intake.

#### Measurement of blood ethanol concentration

In 27 rats (14 sham- and 13 RMTg-lesioned) that had received 8 weeks of IEA, blood ethanol concentration (BECs) were measured after voluntary ethanol intake during the IEA. However, 1 sham-lesioned rat was eliminated due to problems with sample preparation, resulting in a final sample size of 13 sham- and 13 RMTg-lesioned rats for BEC determination. Specifically, tail vein blood was collected after the first 30 min of ethanol access, the interval during which previous results suggest intake rates are highest (Carnicella et al. 2009; Haack et al. 2014). Blood plasma was isolated from samples by perchloric acid precipitation and centrifugation at 2000 rpm for 5 min. NAD-NADH enzyme spectrophotometric method was used to measure BECs (Weiss et al. 1993; Zapata et al. 2006).

#### Operant responding for ethanol

Operant responding for ethanol was investigated in a subset of rats (15 sham- and 14 RMTg-lesioned) provided IEA. Five sham- and three RMTg-lesioned rats were eliminated from analysis since they failed to reach a threshold criterion of 0.3 g/kg/h of ethanol intake in the operant paradigm (Simms et al. 2010; Bertholomey et al. 2013). Data from two other RMTg-lesioned rats were excluded due to improper lesion placement. In the end, data from 10 sham- and 9 RMTg-lesioned rats were included in the final analysis.

Training occurred in 8 Med Associates chambers (St. Albans, VT), enclosed in sound-attenuating cabinets and equipped with ventilation fans. Each chamber contained a recessed magazine where 20 % ethanol could be delivered via a programmable syringe pump. The magazine was flanked by two retractable levers, and illuminated cue lights were positioned above each lever. The right lever always served as the active lever. Responding on the active lever extinguished the cue light, retracted the lever, and delivered 0.1 ml ethanol into the magazine. After a 5-s time-out period, the lever was extended and the cue light again illuminated. The lever located to the left of the magazine served as the inactive lever. Responding on the inactive lever had no programmed consequences. In early training sessions, only the active lever was present and every lever response was reinforced (i.e. FR1 schedule). After an initial overnight session, rats were trained daily in 1-h sessions until they responded at stable levels (less than 20 % variability between 2 sessions). Rats reached this criterion after 3.9 ± 0.4 (sham-lesioned) and 3.8 ± 0.4 sessions (RMTg-lesioned rats; no significant difference, *p* = 0.58). The response requirement was then increased to an FR3 schedule (i.e., every third lever press was reinforced). Rats were trained on this paradigm for 2 sessions, after which the inactive lever was introduced as a measure of non-specific responding. All rats were trained in this final paradigm for 7 sessions. The responses on the active lever were averaged across the last 3 sessions for each rat as a final measure of operant responding.

#### Extinction and reinstatement of operant ethanol seeking

Extinction and reinstatement were tested in the same group of rats trained in operant responding for ethanol (10 sham- and 9 RMTg-lesioned). Extinction sessions were identical to operant training sessions except that the syringe containing ethanol was removed from the syringe pump. Thus, responding on the active lever resulted in retraction of the lever, extinguishing of the cue light, and activation of the syringe pump, but no ethanol delivery. Extinction sessions were conducted daily. Once extinction responding declined to 15 or fewer active lever presses per session for 3 consecutive sessions, rats were tested for yohimbine-induced reinstatement.

Yohimbine (2 mg/kg, IP) or vehicle solution (distilled water) was administered 30 min prior to measurement of operant responding in a 90-min extinction session. A longer extinction session (vs. 60 min training sessions) was used to ensure reliable and robust yohimbine-induced reinstatement in control rats (Gill et al. 2013). Each animal received two injections of yohimbine and two injections of the vehicle solution, with the injection schedule counter-balanced across rats. Each test session that included an injection was separated by an extinction session without injection to ensure reinstated responding was reduced to criterion rates of extinction responding. Responses were then averaged for the two reinstatement tests for each rat.

#### Ethanol-induced CTA

A total of 29 rats (15 sham- and 14 RMTg-lesioned) were conditioned in a CTA paradigm (Rinker et al. 2011). Data from 1 sham- and 2 RMTg-lesioned rats were excluded due to mortality (1 sham-lesioned) and misplaced lesions (2 RMTg-lesioned), resulting in inclusion of data from 14 sham- and 12 RMTg-lesioned rats. Rats were first water deprived for 24 h. Over the next 3 days, rats received 20-min daily access to tap water in the home cage. These 20-min daily access periods were the only time in which fluids were provided throughout CTA training and testing.

CTA training was initiated on the following day. Rats received access to saccharin (0.125 % in tap water) for 20 min in the home cage. Consumption was measured, and rats were immediately divided into 4 groups matched for levels of intake: sham-vehicle, sham-ethanol, RMTg lesioned-vehicle and RMTg lesioned-ethanol. Rats were then injected with 1.5 g/kg body weight of 20 % ethanol (*v*/v, IP) or an equivalent volume of vehicle (saline). On each of the following 2 days, rats again received 20-min access to tap water in their home-cages. This 3-day cycle (saccharin access followed by injection, followed by 2 days of water access without injection) was repeated a total of 3 times (trials 1–3).

CTA was then extinguished through administration of additional 3-day cycles in which there was no injection following each period of saccharin access. These extinction trials continued until saccharin consumption returned to pre-injection baseline levels (trials 4–14). During trials 4 and 5 (the first two extinction trials), we noted marked variability in rats’ latency to initiate lick after given access to saccharin. Thus, starting with the sixth trial, the latency to initiate lick was measured with a timer by an observer who was blind to the treatment condition.

#### Verification of lesions

Rats were deeply anesthetized with sodium pentobarbital (140 mg/kg) and transcardially perfused with saline, followed by 4 % formaldehyde. The brains were removed, cryoprotected and then cut on a freezing microtome (45-μm thickness). Brain sections were immunostained for neuronal nuclei (NeuN) (Furlong and Carrive 2007). Briefly, sections were prepared in 50 % ethanol, 3 % H_2_O_2_, and 5 % normal horse serum, and then incubated in Mouse anti-NeuN (1:5000 for 24-h; Merck-Millipore), followed by anti-mouse IgG (1:1000 for 24-h; Vector Laboratories) and Vectastain ABC reagent (1:750 for 2-h; Vector Laboratories). Finally, a black reaction product was created using a nickel-intensified diaminobenzidine (DAB) reaction. Sections were then mounted onto gelatin-coated slides, dried, cleared in xylene, and cover slipped with DPX mounting medium. Lesions were verified using a light microscope and plotted on templates modified from a reference rat brain atlas (Paxinos and Watson 2007).

Though damage was largely confined to the RMTg, some lesion sites encroached upon nearby structures including the interpeduncular nucleus (IPN), median raphe (MRN), and posterior VTA (pVTA). To determine if damage to these structures affected voluntary ethanol intake in the IEA paradigm, we quantified the damage to IPN, MRN, and pVTA in each RMTg-lesioned rat. The damage to each of these structures was assessed by visual inspection and was scored as 0 (no damage), 25, 50, 75, and 100 % (complete ablation) in each hemisphere. Scores for each hemisphere were averaged to produce a single estimate of damage to the IPN, MRN and pVTA in each RMTg-lesioned rat. Rats were divided into groups by performing a median split and ethanol consumption in the IEA paradigm was statistically compared between the groups (“small” and “large” lesion groups, see Table 2). Because five of six RMTg-lesioned rats used in the CTA paradigm had very little damage outside the RMTg, this analysis was not carried out for the CTA experiment.

**Table 2:** Quantification of ancillary damage to structures close to the RMTg in RMTg-lesioned rats

Brain structure	Small lesion group (% volume lesioned **±** SEM)	Large lesion group (% volume lesioned **±** SEM)	Ethanol intake—small lesion group (g/kg/24 h)	Ethanol intake—large lesion group (g/kg/24 h)	*p* value
IPN	7 ± 1	37 ± 5	6.3 ± 1.0	5.5 ± 1.2	0.11
MNR	9 ± 2	30 ± 5	6.2 ± 1.1	6.1 ± 1.4	0.50
pVTA	23 ± 2	39 ± 2	6.8 ± 1.1	5.5 ± 1.2	0.46

Rows indicate each brain structure analyzed. The first two columns indicate the volume of each brain structure lesioned in “small” and “large” lesion groups (determined by median split). Ethanol intake for each of the two groups is indicated for the small and large lesions groups in columns 3 and 4, respectively. The final column indicates significance values comparing levels of ethanol intake; there were no significant differences between any of the small and large lesions groups

#### Statistical analyses

Lesion effects on BEC after IEA and operant ethanol self-administration were analyzed using *t* tests. Differences in “small” and “large’ lesion groups with regard to ethanol consumption in the IEA paradigm were analyzed using *t* test. Voluntary ethanol consumption, ethanol preference, water intake, and total fluid intake during IEA were analyzed using two-way RM ANOVA (factors of lesion and drinking session). Taste preference, extinction of ethanol-seeking, and yohimbine-induced reinstatement were also analyzed using two-way RM ANOVA (factors of lesion and: tastant concentration, extinction session and drug, respectively). Analysis of CTA data was carried out separately for acquisition (trials 1–4) and extinction (trials 5–14), using three-way RM ANOVA (factors of lesion, drug, and trial) for each of these stages. JMP Pro 11 (SAS Institute Inc., Cary, NC) was used to carry out analyses. For all tests, a criterion of *p* < 0.05 was used to establish significant differences.

## Results

### Voluntary ethanol consumption

**Fig. 1:**
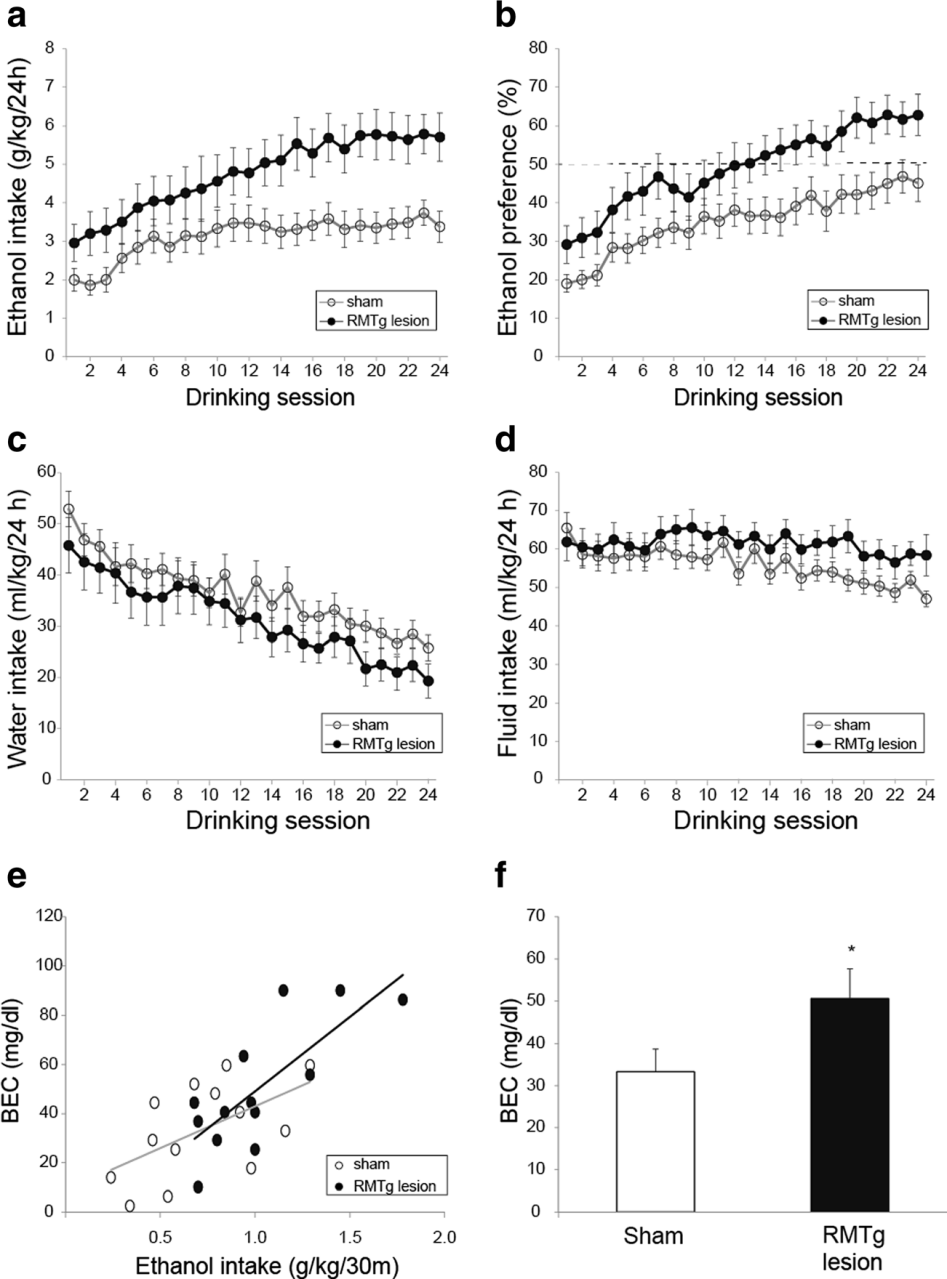
RMTg lesion increases voluntary consumption in an intermittent ethanol access (IEA) paradigm. **a** RMTg-lesioned rats voluntarily consumed significantly more 20 % ethanol as compared to sham-lesioned animals. *Open symbols* represent data for sham-lesioned rats, and *closed circles* represent data for RMTg-lesioned rats in this and all subsequent figures unless otherwise mentioned. *Symbols* depict mean ± SEM. **b** RMTg-lesioned rats had a higher preference for ethanol relative to sham-lesioned animals. **c** Water intake decreased progressively in both groups. There were no significant differences between sham- and RMTg-lesioned rats. **d** Total fluid intake did not differ between sham- and RMTg-lesioned rats. **e** Blood ethanol concentration (*BEC*) was significantly correlated with ethanol intake in the first 30 min for both sham- and RMTg-lesioned rats. *Gray line* shows linear fit for sham-lesioned rats, and *black line* shows linear fit for RMTg-lesioned rats. **f** BEC for RMTg-lesioned after voluntary intake was significantly higher than that measured for sham-lesioned animals

Intermittent access to ethanol increased ethanol intake in both sham- and RMTg-lesioned animals over the study period of 8 weeks (Fig. 1a; significant main effect of drinking session, *F*(3.5, 179.7) = 13.2, *p* < 0.0001). However, RMTg-lesioned rats drank more ethanol than the sham-lesioned rats (significant main effect of lesion, *F*(1.51) = 6.9, *p* < 0.05 but no significant interaction of lesion and drinking session, F(3.5, 179.7) = 1.9, *p* = 0.12). RMTg-lesioned rats also had higher preference for ethanol relative to sham-lesioned rats (Fig. 1b; significant main effect of lesion, *F*(1.51) = 5.6, *p* < 0.05, and significant main effect of drinking session, *F*(4.3, 218.4) = 24.6, *p* < 0.0001, no significant interaction of lesion and drinking session, *F*(4.3, 218.4) = 0.8, *p* = 0.53).

Water intake declined progressively for both sham- and RMTg-lesioned groups during the 8 weeks of IEA (Fig. 1c; significant main effect of drinking session, *F*(5.1, 258.5) = 20.3, *p* < 0.0001). RMTg lesion had no significant effect on water intake (no significant main effect of lesion, *F*(1.51) = 1.8, *p* = 0.18, and no significant interaction of lesion and drinking session, *F*(5.1, 258.5) = 0.5, *p* = 0.8). Further, total fluid intake did not differ between sham- and RMTg-lesioned rats (Fig. 1d; no significant main effect of lesion, *F*(1.51) = 2.1, *p* = 0.16 and no significant interaction of drinking session and lesion, *F*(4.1, 207.3) = 1.2, *p* = 0.31). Also, RMTg lesion did not alter weekly food intake (150.6 ± 4.1 g/week and 158.1 ± 7.7 g/week for sham- and RMTg-lesioned rats, respectively; no significant main effect of lesion, *F*(1.25) = 0.6, *p* = 0.46, and no significant interaction of lesion and time, *F*(4.5, 112.5) = 2, *p* = 0.09).

BEC analyzed from tail vein blood obtained after the first 30 min of access to ethanol in the IEA paradigm revealed a significant correlation between BEC and ethanol intake normalized to weight (Fig. 1e; *r* ^2^ = 0.31 and *r* ^2^ = 0.60 for sham- and RMTg-lesioned, respectively; *p* < 0.05 for each group, Pearson’s correlation). Mean BEC was significantly higher in RMTg-lesioned rats relative to controls (Fig. 1f; *t* = 1.9, *p* = 0.03).

### Taste preference

**Fig. 2:**
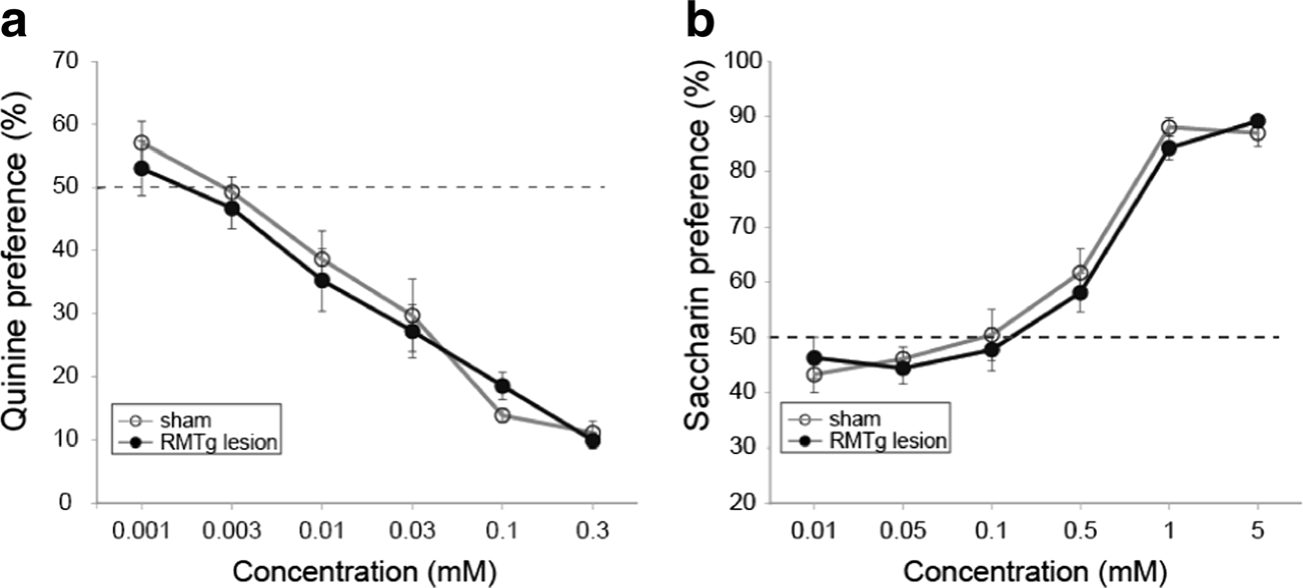
RMTg lesions do not alter saccharin preference or quinine aversion. RMTg lesion did not alter **a** aversion to quinine or **b** preference for saccharin

Ethanol has both bitter and sweet taste components (Scinska et al. 2000; Blizard 2007). To rule out the possibility that higher ethanol intake in RMTg-lesioned rats was due to altered taste preference, we studied quinine (bitter) and saccharin (sweet) preference in sham- and RMTg-lesioned rats. Quinine aversion increased in both sham- and RMTg-lesioned groups with increasing quinine concentration (Fig. 2a; significant main effect of quinine concentration, *F*(3.6, 90.2) = 71.2, *p* < 0.0001). However, RMTg lesion had no significant effect on quinine aversion (no significant main effect of lesion, *F*(1.25) = 0.2, *p* = 0.66 and no significant interaction of lesion and quinine concentration, *F*(3.6, 90.2) = 0.6, *p* = 0.66). Saccharin preference increased with increasing saccharin concentration for both sham- and RMTg-lesioned rats (Fig. 2b; significant main effect of saccharin concentration, *F*(3.6, 91.4) = 114.5, *p* < 0.0001). Again, RMTg lesion had no significant effect on saccharin preference (no significant main effect of lesion, *F*(1.25) = 0.1, *p* = 0.71 and no significant interaction of lesion and saccharin concentration, *F*(3.6, 91.4) = 0.6, *p* = 0.63).

### Operant self-administration of 20 % ethanol

**Fig. 3:**
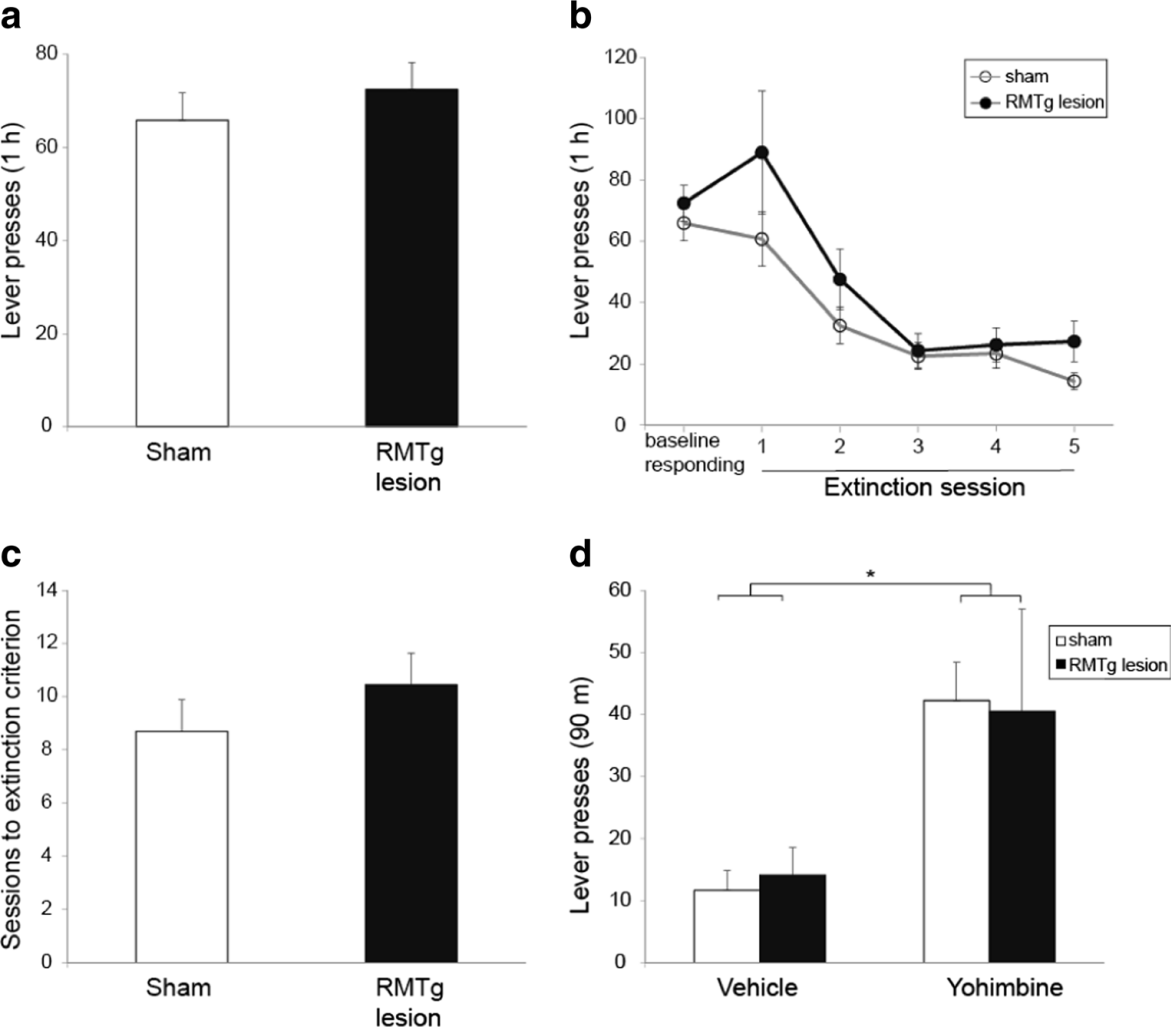
RMTg lesions do not alter operant self-administration of ethanol, extinction of operant responding, or yohimbine-induced reinstatement of ethanol-seeking. **a** RMTg lesion did not significantly change operant responding for 20 % ethanol, **b** the rate of operant extinction, **c** the mean number of extinction sessions required to reach the extinction criterion, **d** Yohimbine caused robust reinstatement in both sham- and RMTg-lesioned rats. *Asterisk* indicates the main effect of drug

RMTg- and sham-lesioned rats did not differ in the average number of active lever presses during the last 3 sessions of operant training (Fig. 3a; *t* = −0.8, *p* = 0.21), nor were there differences in inactive lever presses between the two groups (data not shown, *t* = 0.9, *p* = 0.17). Mean ethanol intake during the last 3 sessions did not differ between the two groups (0.59 ± 0.05 vs. 0.66 ± 0.06 g/kg/24 h for sham- and RMTg-lesioned rats, respectively, *t* = −0.95, *p* = 0.17).

### Extinction and yohimbine-induced reinstatement of ethanol-seeking

Extinction resulted in a sharp decline in responding on the active lever (Fig. 3b; significant main effect of extinction session, *F*(2.6, 44.9) = 24.2, *p* < 0.0001). However, RMTg lesion had no significant effect on the rate of extinction (no significant main effect of lesion, *F*(1.17) = 2.5, *p* = 0.13 and no significant interaction of lesion and extinction session, *F*(2.6, 44.9) = 1, *p* = 0.39). We also analyzed the average number of extinction sessions required to reach the extinction criterion. There was no statistically significant difference between sham- and RMTg-lesioned rats (Fig. 3c; *t* = −1, *p* = 0.15). Finally, RMTg lesion had no significant effect on inactive lever presses during extinction (data not shown; no significant main effect of lesion, *F*(1.17) = 1.09, *p* = 0.31, and no significant interaction of lesion and extinction session, *F*(5.85) = 0.2, *p* = 0.96).

Yohimbine administration reinstated operant responding in both sham- and RMTg-lesioned rats (Fig. 3d; significant main effect of drug, *F*(1.17) = 19.5, *p* < 0.001). RMTg lesion did not alter yohimbine-induced reinstatement of ethanol-seeking (no significant main effect of lesion, *F*(1.17) = 0.001, *p* = 0.97 and no significant interaction of lesion and drug *F*(1.17) = 0.1, *p* = 0.75). Neither yohimbine administration nor RMTg lesion had significant effects on inactive lever presses during reinstatement sessions (data not shown; no significant main effect of drug, *F*(1.17) = 2.7, *p* = 0.12; no significant main effect of lesion, *F*(1.17) = 1.9, *p* = 0.18; no significant interaction of lesion and drug, *F*(1.17) = 0.6, *p* = 0.43).

### Ethanol-induced CTA

#### Acquisition of ethanol-induced CTA

**Fig. 4:**
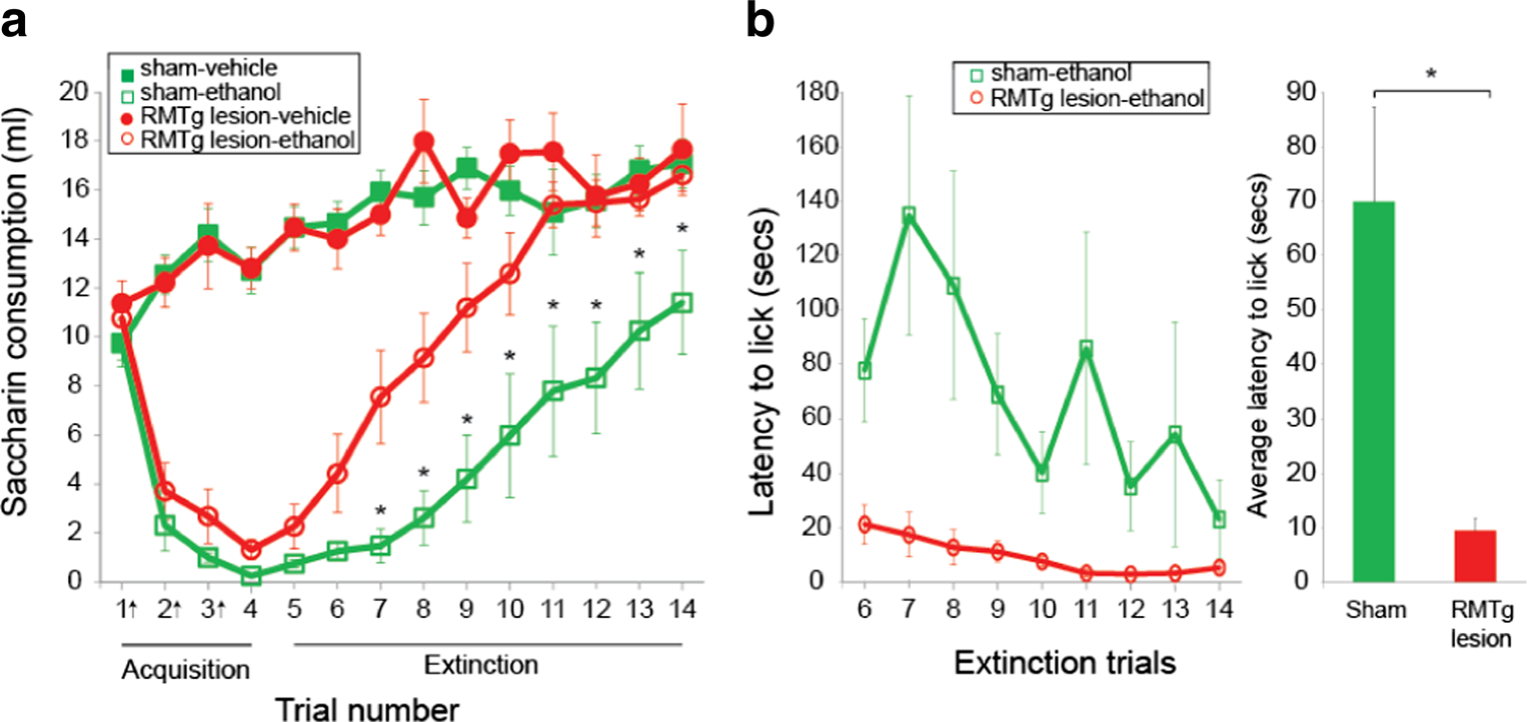
RMTg lesions accelerate extinction of CTA. **a** Sham-lesioned rats are shown as *squares*, and RMTg-lesioned rats are shown as *circles*. *Open* and *closed symbols* indicate treatment with ethanol and saline (vehicle), respectively. *Trials 1*–*4* are acquisition trials and *trials 5*–*14* are extinction trials. *Arrows* (*x axis*) indicate the trials in which saccharin access was paired with ethanol injection. *Asterisks* indicate significant differences between sham- and RMTg-lesioned groups who received ethanol injection (*p* < 0.05). **b** RMTg lesion reduced the latency to initiate lick during extinction trials. *Bar graph* shows the average latency to initiate lick (seconds) for *trials 6*–*14*. RMTg-lesioned rats had significantly lower latency to initiate lick compared to sham-lesioned rats

Ethanol (1.5 g/kg, IP) conditioned a robust conditioned taste aversion in both sham- and RMTg-lesioned rats (Fig. 4a, trials 1–4; significant main effect of drug, *F*(1.22) = 129.5, *p* < 0.0001; significant main effect of trial, *F*(3.66) = 23.1, *p* < 0.0001 and significant interaction of drug and trial, *F*(3.66) = 73.8, *p* < 0.0001) as indicated by a reduction in saccharin consumption for both groups after pairing with ethanol injection. The acquisition of CTA did not differ between sham- and RMTg-lesioned rats (no significant interaction of lesion and drug, *F*(1.22) = 0.6, *p* = 0.48; no significant main effect of lesion, *F*(1.22) = 1, *p* = 0.3 and no significant 3-way interaction of lesion, drug, and trial, *F*(3.66) = 0.9, *p* = 0.46).

#### Extinction of ethanol-induced CTA

The rate of CTA extinction was dependent on lesion (Fig. 4a, trials 5–14; significant main effect of lesion, *F*(1.22) = 6.9,*p* < 0.05 and significant interaction of lesion and drug, *F*(1.22) = 5.6, *p* < 0.05; RMTg-lesioned rats showed significantly more rapid extinction than sham-lesioned animals). There was no significant 3-way interaction of lesion, drug, and trial, *F*(4.4, 97.8) = 1.1, *p* = 0.37. Post hoc tests revealed that RMTg-lesioned rats injected with ethanol consumed significantly more saccharin than sham-lesioned rats injected with ethanol from trial 7–14 (*p* < 0.05).

We also analyzed the latency to initiate licking during trials 6–14 (extinction trials). A two-way RM ANOVA revealed that RMTg-lesioned rats initiated saccharin consumption at significantly shorter latency than sham-lesioned animals (Fig. 4b; significant main effect of lesion, *F*(1.11) = 9.9, *p* < 0.01). Average latency to initiate licking across trials 6–14 was significantly shorter in RMTg-lesioned rats (Fig. 4b; *t* = 3.4, *p* < 0.05).

### Histological confirmation of lesions for RMTg

**Fig. 5:**
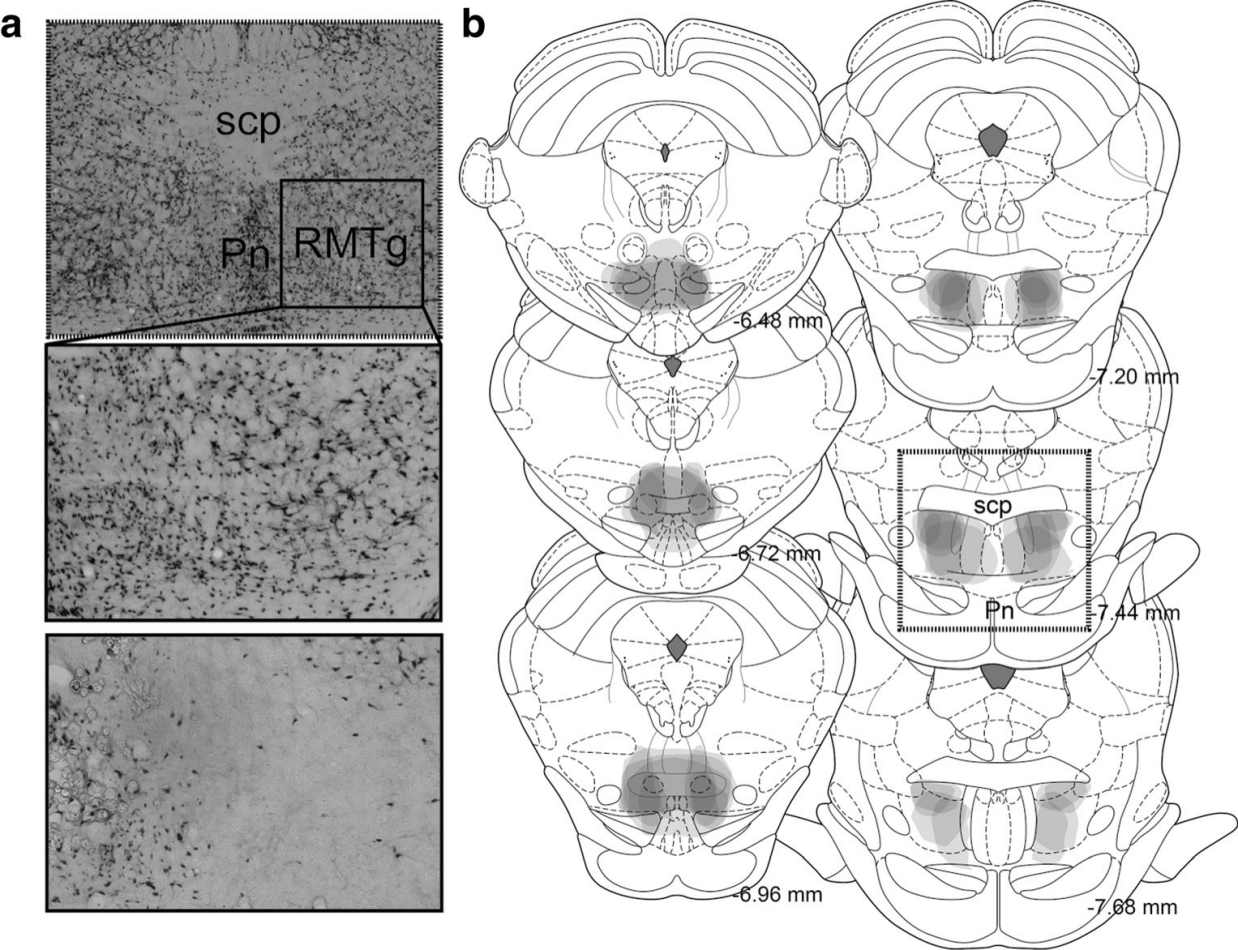
RMTg lesion placements. **a** Representative photomicrograph of RMTg lesion. The *upper* and *middle panels* show sections from a sham-lesioned rat while the *lower panel* demonstrates a section from a RMTg-lesioned rat. Note the considerably reduced NeuN staining in the RMTg-lesioned rat. **b** Excitotoxic lesion sites for 6 representative rats are overlaid such that the areas with the darkest shading have the maximum damage. Damage was restricted to RMTg in most cases; however, there was some damage to adjacent areas including the median raphe and interpeduncular nucleus in some rats. The anterior-posterior coordinates relative to bregma are shown to the *right* of each coronal section (*scp* superior cerebellar peduncle, *Pn* pontine nuclei)

Lesions were largely confined to the RMTg (Fig. 5). In a few cases, damage encroached upon neighboring structures including the IPN, MRN, and pVTA.

To determine if damage to these areas contributed to increased ethanol intake in RMTg-lesioned rats, we quantified the damage for each of the areas for each RMTg-lesioned rat tested in the IEA paradigm. We then compared intake in rats with low and high levels of damage (determined through a median split); we found no significant differences in these groups with respect to ethanol intake in the IEA paradigm (Table 2).

## Discussion

In the current set of experiments, we studied the effects of RMTg lesion on voluntary ethanol consumption, operant-ethanol self-administration, yohimbine-induced reinstatement, and ethanol-induced CTA. Our findings indicate that the RMTg plays an important role in regulating voluntary ethanol intake, possibly by accelerating extinction of ethanol-induced aversion. RMTg-lesioned rats voluntarily consumed more ethanol in the IEA paradigm as compared to sham-lesioned animals. In addition, RMTg lesions caused more rapid extinction of ethanol-induced CTA. RMTg lesions did not significantly change operant ethanol self-administration, extinction of ethanol-seeking or yohimbine-induced reinstatement of ethanol-seeking. We discuss the implications of these findings in more detail below.

### Effects of RMTg lesion on voluntary ethanol consumption and CTA

RMTg-lesioned rats showed increased ethanol consumption and ethanol preference in the IEA paradigm as compared to sham-lesioned rats (Fig. 1a, b). Altered taste processing is unlikely to have influenced ethanol consumption, given that there were no significant differences in quinine aversion and saccharin preference between RMTg- and sham-lesioned rats (Fig. 2a, b).

Accelerated extinction of ethanol-induced CTA could contribute to increased ethanol consumption in RMTg-lesioned rats. Although ethanol injection conditioned a robust CTA to saccharin in both sham- and RMTg-lesioned groups, RMTg lesions accelerated the rate at which rats returned to pre-injection levels of saccharin consumption (Fig. 4a). RMTg-lesioned rats also initiated licking at the saccharin bottle faster than sham-lesioned rats during extinction trials, again indicative of faster CTA extinction (Fig. 4b).

The RMTg has previously been implicated in regulating aversion learning, including that driven by drug rewards (Stamatakis and Stuber 2012; Jhou et al. 2013). Thus, it may be possible that elevated consumption in RMTg-lesioned rats resulted from an attenuation in the persistence of ethanol-induced aversive learning. Further experiments are needed to directly test if the increased ethanol consumption in RMTg-lesioned rats is a learned behavior caused by acceleration of extinction of ethanol-induced CTA. It is interesting to note that ethanol administration activates RMTg neurons in alcohol non-preferring rats (sNP) but not alcohol preferring rats (sP); the latter have both increased voluntary ethanol intake and attenuated ethanol-induced CTA (Brunetti et al. 2002; Melis et al. 2014).

Several caveats attend to our interpretation of these results. One limitation of the current experimental design is that all rats tested in the CTA paradigm had prior ethanol experience in the IEA paradigm. Thus, differential ethanol exposure occurring during IEA may have contributed to apparently lesion-dependent differences in behaviors downstream of voluntary intake, particularly CTA. In addition, processes underlying accelerated extinction of CTA in RMTg-lesioned rats are not well-defined, and could, for instance, have resulted from accelerated learning about the safety of the ethanol-associated taste. And while the balance of available evidence suggests a primary role for the RMTg in mediating aversion learning, our findings do not rule out a role for this brain region in mediating ethanol-induced reward (or even physiological tolerance to ethanol, which might lead to higher levels of voluntary intake). Another important consideration while interpreting our CTA results is that we used a relatively high dose of ethanol (1.5 g/kg) over 3 conditioning sessions. Thus, it is possible that we may have encountered floor effects. Conditioning with a lower ethanol dose or fewer conditioning sessions could have revealed differences in the acquisition of CTA between the two groups. Additional experiments addressing these issues will be useful in further characterizing our results.

### Neural circuits underlying ethanol intake and CTA

Our current results are consistent with and extend previous findings in which we showed that lesions of the LHb, which provides a major afferent input to the RMTg, increase voluntary ethanol consumption and attenuate ethanol-induced CTA (Haack et al. 2014). The LHb inhibits VTA DA neurons through a disynaptic pathway involving the RMTg, in which the LHb sends a glutamatergic projection to the RMTg, which in turn sends a primarily GABAergic inhibitory projection to VTA DA neurons (Jhou et al. 2009b; Kaufling et al. 2009, 2010; Hong et al. 2011).

The LHb-RMTg-VTA pathway has been implicated both in mediating acute behavioral responses to aversive stimuli (e.g., avoidance) and in aversion learning. LHb and RMTg neurons are activated in response to a range of conditioned and unconditioned aversive stimuli (Wirtshafter et al. 1994; Timofeeva and Richard 2001; Matsumoto and Hikosaka 2009; Hong et al. 2011; Brown and Shepard 2013). Combined functional and anatomical studies have shown that RMTg neurons activated by aversive stimuli specifically include those that receive afferents from the LHb and project to VTA DA neurons (Jhou et al. 2009a). Activation of the LHb-RMTg projection is negatively reinforcing and produces active, passive, and conditioned avoidance (Stamatakis and Stuber 2012).

Drug taking is governed by the relative balance of drug-induced reward and aversion, in which the aversive effects of drugs of abuse serve as limiting factors in regulating intake (Riley 2011). Given the role of the LHb-RMTg projection in processing aversive stimuli and promoting avoidance behaviors, this pathway is well-suited to play a central role in mediating drug-induced aversion learning that impacts voluntary drug intake. Recent findings suggest that this anatomical pathway indeed plays this role. Cocaine excites LHb neurons, including those that project to the RMTg, and potentiates LHb to RMTg synapses (Maroteaux and Mameli 2012). Increased firing of the LHb neurons in turn causes cocaine-evoked negative symptoms and cocaine-induced avoidance behaviors; optogenetic inhibition of the LHb to RMTg terminals during cocaine-induced excitation abolishes this avoidance behavior (Jhou et al. 2013; Meye et al. 2015). These results implicate activity in the LHb-RMTg pathway in driving drug-induced aversive conditioning.

CTA requires signaling through a pathway that includes but is not limited to the nucleus tractus solitarius (NTS), parabrachial nucleus (PBN), insular cortex (IC) and nucleus accumbens (Nacc) (Yamamoto et al. 1994; Thiele et al. 1996; Ramirez-Lugo et al. 2007). In addition, it has been shown that ethanol-induced CTA is dependent on signaling through D1 and D2 receptors (Risinger et al. 1999). Interestingly, ethanol-induced CTA appears not to require signaling through the area postrema, which is required for many other forms of CTA (Stewart et al. 1988). The LHb-RMTg pathway is well-positioned to interact with this network, as the NTS and PBN, which carry ascending sensory information, project directly to the RMTg (Jhou et al. 2009b; Yetnikoff et al. 2015). Further, the IC, which is critical for expression of CTA (Schier et al. 2016) and the NAcc, which is crucial for taste memory formation (Ramirez-Lugo et al. 2007) also projects to the LHb and RMTg (Vadovicova 2014; Yetnikoff et al. 2015). Finally, given the dependence of ethanol-induced CTA on DA signaling, it is possible that the LHb-RMTg pathway regulates CTA through modulation of DA neuron firing.

### Effects of RMTg lesion on operant ethanol self-administration, extinction, and yohimbine-induced reinstatement

We previously showed that LHb lesion elevated operant responding for ethanol (Haack et al. 2014). Somewhat surprisingly, RMTg lesion had no effect on this behavior (Fig. 3a), suggesting that operant ethanol-seeking behaviors are differentially dependent on the LHb and RMTg. Operant self-administration examines appetitive or “seeking” behavior, whereas free access to the reinforcer in the home-cage examines consummatory or “taking” behavior. The neural substrates of these two behaviors are known to be different. For example, systemic administration of a DA D2 receptor antagonist reduces ethanol seeking, but not ethanol taking; conversely, administration of naltrexone reduces ethanol taking without affecting ethanol seeking (Brown et al. 1982; Czachowski et al. 2001; Czachowski et al. 2002). These results are consistent with other studies suggesting a relatively specific role for opioid signaling in predominantly modulating consummatory behaviors, while dopamine signaling predominantly regulates appetitive behaviors (Kelley et al. 2005). Thus, there is precedent for distinct mechanisms underlying these two categories of behaviors, and it is possible that the LHb may serve as a convergence point for consummatory and appetitive behaviors, whereas the RMTg selectively modulates the former. That said, the neural pathways that would differentially involve the LHb and RMTg remain unclear, particularly given the strong projection from the former to the latter. One possibility is that the direct projection from the LHb to the VTA (Goncalves et al. 2012) is important in mediating LHb lesion effects on operant ethanol-seeking, given that VTA is critical for ethanol-seeking (Czachowski et al. 2012).

It is also possible that the RMTg contributions to appetitive behaviors are significant, but were simply not detected in the present experiments. Notably, a recent report showed that RMTg activity regulated extinction of an appetitive behavior, cocaine-seeking (Huff and LaLumiere 2014). Methodological differences (permanent excitotoxic lesions vs. acute infusion of AMPA potentiator) and the drug tested (ethanol vs. cocaine) may have contributed to these divergent results.

Similarly, while we and others have shown that LHb lesion blocks yohimbine-induced reinstatement of drug-seeking (Gill et al. 2013; Haack et al. 2014), RMTg lesions had no effect on this behavior (Fig. 3d). LHb efferents targeting structures other than the RMTg may critically regulate this behavior. The medial portion of the LHb sends direct projections to the serotonin-rich dorsal raphe (DR) and median raphe nuclei (MRN) (Herkenham and Nauta 1979; Sego et al. 2014). Manipulations of the DR and MRN alter yohimbine-induced reinstatement (Le et al. 2013) and other stress-induced behavioral responses (Maier and Watkins 2005; Dayan and Huys 2009; Cools et al. 2011). Direct projections of the LHb to DR and/or MRN (or another projection target) may thus mediate LHb effects on yohimbine-induced reinstatement.

A limitation of the current results is that RMTg lesion effects on operant ethanol-seeking and yohimbine-induced reinstatement of ethanol-seeking were tested in alcohol-experienced rats. We cannot rule out the possibility that RMTg lesion effects on these ethanol-directed behaviors may differ in ethanol-naïve rats.

## Conclusions

Our findings show that the RMTg plays an important role in regulating voluntary ethanol intake, possibly by mediating aversive conditioning to ethanol. Lesion of the RMTg increased voluntary ethanol consumption and accelerated extinction of ethanol-induced CTA. Surprisingly, we found that the RMTg (unlike the LHb) plays little apparent role in regulating ethanol-directed operant behavior and yohimbine-induced reinstatement of ethanol seeking.
